# Supplementary material for: Splice-Junction-Based Mapping of Alternative Isoforms in the Human Proteome
Source: Cell Rep. Author manuscript; Available in PMC 2020 Jan 15. (PMC6961840; doi:10.1016/j.celrep.2019.11.026)
Supplement: 2 [file NIHMS1546469-supplement-2.zip › DF1/PXD010154/Thyroid/Thyroid_3_TG_VTGGQPACERPQLWQTIQTQGHFQLQLPPGK.pdf]

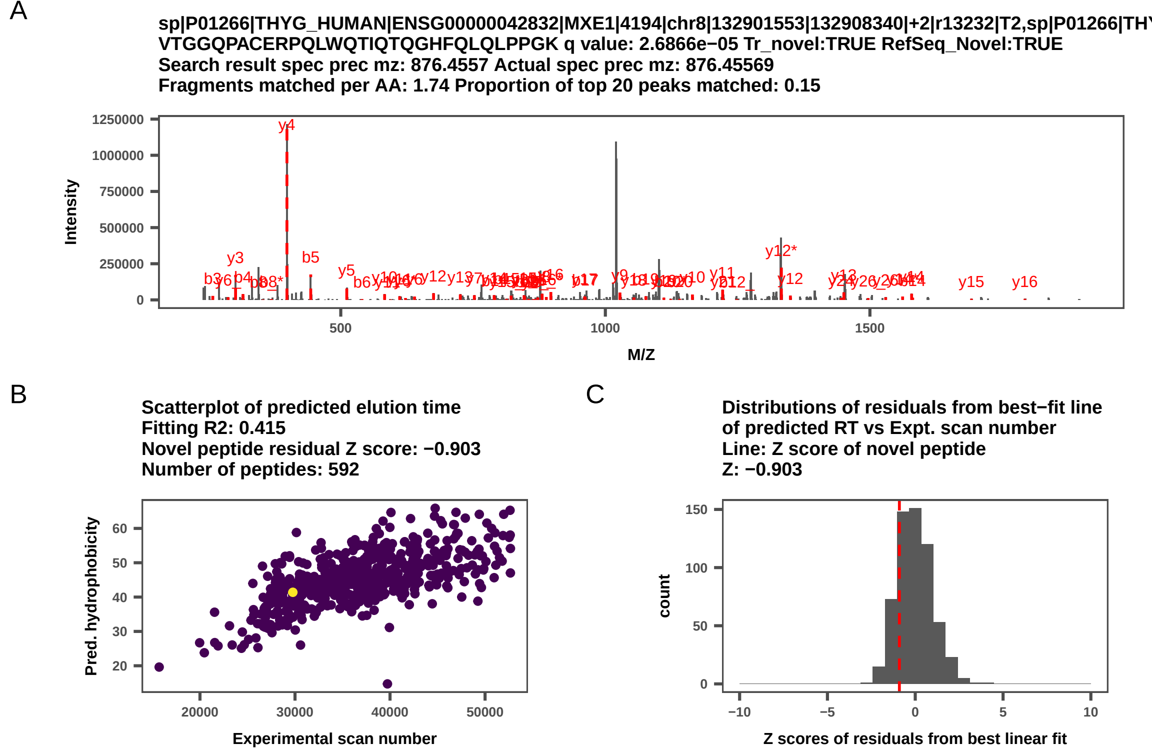
A
sp|P01266|THYG_HUMAN|ENSG00000042832|MXE1|4194|chr8|132901553|132908340|+2|r13232|T2,sp|P01266|THY
VTGGQPACERPQLWQTIQTQGHFQLQLPPGK q value: 2.6866e−05 Tr_novel:TRUE RefSeq_Novel:TRUE
Search result spec prec mz: 876.4557 Actual spec prec mz: 876.45569
Fragments matched per AA: 1.74 Proportion of top 20 peaks matched: 0.15
Intensity
M/Z
B
Scatterplot of predicted elution time
Fitting R2: 0.415
Novel peptide residual Z score: −0.903
Number of peptides: 592
Pred. hydrophobicity
Experimental scan number
C
Distributions of residuals from best−fit line
of predicted RT vs Expt. scan number
Line: Z score of novel peptide
Z: −0.903
count
Z scores of residuals from best linear fit
